# The use of 5-fluorouracil-loaded nanobubbles combined with low-frequency ultrasound to treat hepatocellular carcinoma in nude mice

**DOI:** 10.1186/s40001-017-0291-8

**Published:** 2017-11-21

**Authors:** Qiaoya Li, Hongyang Li, Chengjun He, Zhouhong Jing, Changan Liu, Juan Xie, Wenwen Ma, Huisheng Deng

**Affiliations:** 1 Department of Geriatrics, The First Affiliated Hospital of Chongqing Medical University, No. 1, Youyi Road, Chongqing, 400016 People’s Republic of China; 2 Department of Hepatobiliary Surgery, The Second Affiliated Hospital of Chongqing Medical University, Chongqing, 400010 People’s Republic of China

**Keywords:** Low-frequency ultrasound, Hepatocellular carcinoma, 5-FU-loaded nanobubbles, Cavitation, Anti-tumor effects

## Abstract

**Objective:**

This study aimed to investigate the therapeutic effects of 5-fluorouracil (5-FU)-loaded nanobubbles irradiated with low-intensity, low-frequency ultrasound in nude mice with hepatocellular carcinoma (HCC).

**Methods:**

A transplanted tumor model of HCC in nude mice was established in 40 mice, which were then randomly divided equally into four groups: group A (saline), group B (5-FU-loaded nanobubbles), group C (5-FU-loaded nanobubbles with non-low-frequency ultrasound), and group D (5-FU-loaded nanobubbles with low-frequency ultrasound). The tumor size in each mouse was observed via ultrasound before and after the treatments. Inhibition of the tumor growth in each group was compared, and survival curves were generated. Tumor tissues were removed to determine the apoptotic index using the TUNEL method and quantitative analysis. Tumor tissues with CD34-positive microvessels were observed by immunohistochemistry, and the tumor microvessel densities were calculated.

**Results:**

The growth rate of the tumor volumes in group D was significantly slower than that in the other groups, while the tumor inhibition rates and apoptotic index in group D were significantly higher than those of the other groups. The number of microvessels staining positive for CD34 was decreased in group D. Therefore, group D presented the most significant inhibitory effects.

**Conclusions:**

Therefore, 5-FU-loaded nanobubbles subjected to irradiation with low-frequency ultrasound could further improve drug targeting and effectively inhibit the growth of transplanted tumors, which is expected to become an ideal drug carrier and targeted drug delivery system for the treatment of HCC in the future.

## Background

Hepatocellular carcinoma (HCC) is the fifth most common malignant tumor in the world, with an incidence rate in China second only to gastric and esophageal cancers [1, 2]. Due to the insidious onset and rapid progression of HCC, clinical treatment approaches have relatively poor outcomes [3]. With the development of modern imaging techniques, the clinical diagnosis of HCC has become easier. However, most patients have moderate or advanced stage disease when they seek medical care, resulting in limited traditional treatment options [4]. Therefore, searching for a better method for targeted HCC therapy is important, and the identification of an efficient, well-targeted, and safe drug-loading system is urgently required.

**Fig. 1:**
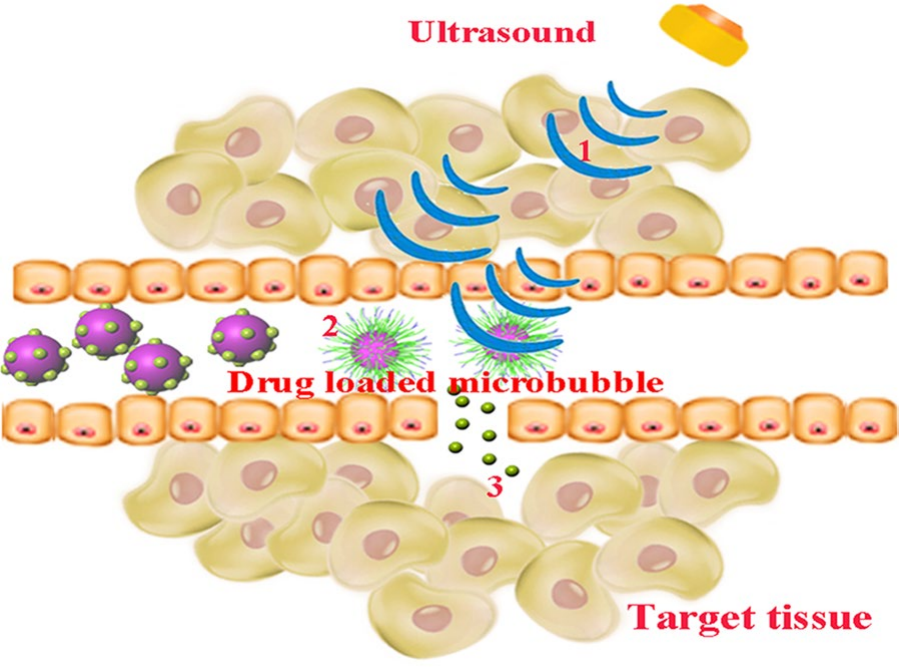
UTMD technology *1* The drug-loaded microbubbles are targeted and irradiated by ultrasound; *2* The drug-loaded microbubbles are disrupted after irradiation, and drugs are released; *3* The drugs are locally released into target tissues and kill the cancer cells

Recently, the targeted destruction of a drug-loaded microbubble by ultrasound (the UTMD technique) has become a hot topic and a new research direction [5–8]. The UTMD technique involves extensively irradiating drug-loaded microbubbles with ultrasonic pulses at the proper energy level when the microbubbles reach the target tissues via the blood stream after peripheral intravenous injection. The “cavitation effect” and “mechanical effect” produced by ultrasonic irradiation can destroy the cell membrane and enhance cell membrane permeability [9–11], promoting the amount of drug that enters the target tissues (Fig. 1).

The UTMD technique has great value in the treatment of tumors because of the superiority of the technique. However, applications of the UTMD technique have relied primarily on ordinary ultrasound rather than focused ultrasound [7, 12, 13], which may decrease the effect of this technique and also damage the surrounding tissues as a result of the wide-range irradiation [12–14]. The lack of safety and the inefficiency of ordinary ultrasound for irradiating the drug-loaded microbubbles have limited the further development of this approach. Although some studies have reported that high-intensity focused ultrasound is a good choice for the UTMD technique [15], the associated high energy may also damage the surrounding normal tissues. Therefore, the search for a safe and efficient technology to disrupt the drug-loaded microbubbles has become a focus of interest for researchers. Considering the current state of research, the Institute of Ultrasound Imaging, Chongqing Medical University has developed a low-intensity and low-frequency focused ultrasound to investigate the feasibility of accurate targeting and disrupting microbubbles with low-frequency ultrasound.

5-Fluorouracil (5-FU) is a cytotoxic anti-tumor drug that has a good therapeutic effect against HCC [16–18]. As revealed by several studies, the targeted destruction of 5-FU-loaded microbubbles by ultrasound is useful for the treatment of tumors [19–21]. In this study, we aimed to promote the safety and efficiency of the targeted ultrasound destruction of 5-FU-loaded microbubbles. Therefore, we used low-frequency focused ultrasound (developed at the Institute of Ultrasound Imaging, Chongqing Medical University) for the targeted destruction of 5-FU-loaded nanobubbles (nanoscale microbubbles) in nude mice with HCC and evaluated the effects of this treatment using various experimental methods and observations. This study may provide a safer, more specifically targeted and more efficient and convenient technology for controlled-release treatments, which is expected to be the foundation for future targeted therapy in HCC.

## Methods

### Experimental materials

#### Nude mice

In this study, forty nude mice of either sex (clean grade, body weight: 20 ± 2 g, 6–8 weeks old) were purchased from the Animal Laboratory of Chongqing Medical University. The protocol was approved by the Animal Research Committee of Chongqing Medical University. The mice were kept separately in specific pathogen-free cages with mixed feed for 2–3 months.

#### Major materials

**Fig. 2:**
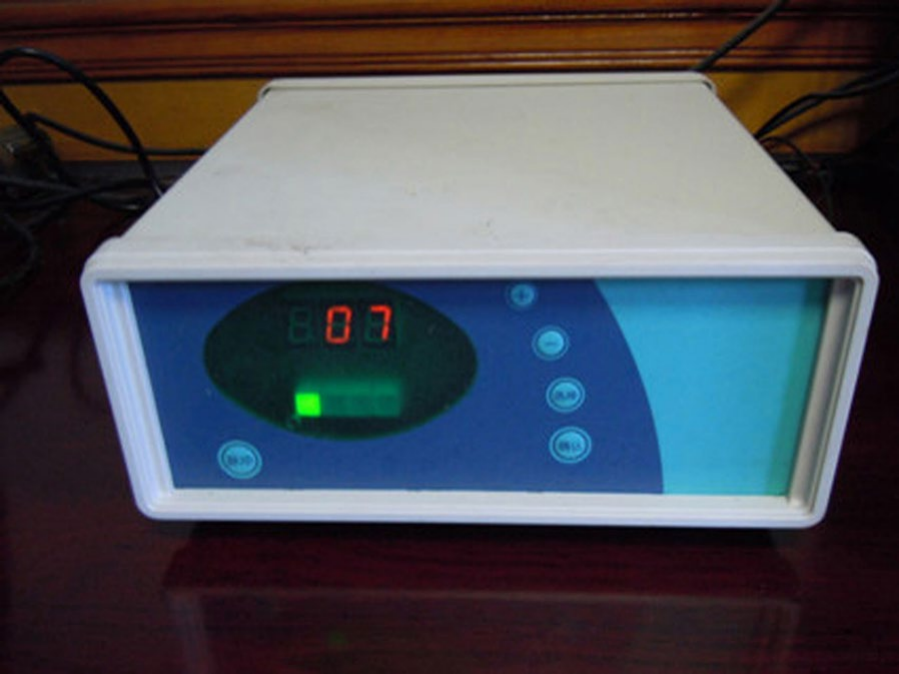
The DZC low-frequency focused ultrasound instrument

A DZC-type low-frequency focused ultrasound instrument was developed and provided by the Ultrasound Research Institute of Chongqing Medical University (Chongqing, China, Fig. 2). Diphenylphosphoryl azide (DPPA), distearoylphosphatidylethanolamine (DSPE) and dipalmitoylphosphatidylcholine (DPPC) were purchased from Sigma (USA); RPMI-1640 and fetal bovine serum were purchased from HyClone (Beijing, China). Mouse anti-human CD34 monoclonal antibodies were purchased from Zhongshan Biological Technology (Beijing, China), and a TUNEL staining kit was purchased from Roche (Basel, Switzerland).

### Methods

#### Transplanted tumor model of HCC in nude mice

The tumor cell lines of HepG2 were cultured in vitro, and only those with an acceptable growth rate were used for this study. The concentration of tumor cells was diluted to approximately 1 × 10^8^/ml using serum-free RPMI-1640 culture medium, and the cell viability was greater than ninety percent as determined by the trypan blue dye exclusion test. Each nude mouse was subcutaneously inoculated with 0.2 ml of the tumor cell suspension in the right flank. After 6 days, the diameters of tumors in the nude mice were 0.5–1.0 cm, and the tumor formation rate in the nude mice was 100%. In this study, forty nude mice were randomly divided equally into four groups: group A (saline), group B (5-FU-loaded nanobubbles), group C (5-FU-loaded nanobubbles with non-low-frequency ultrasound), and group D (5-FU-loaded nanobubbles with low-frequency ultrasound).

#### Synthesis of 5-FU-loaded nanobubbles

**Fig. 3:**
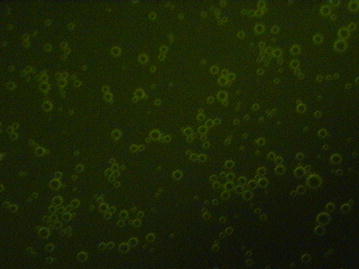
5-FU-loaded nanobubble fluorescence images (original magnification ×400)

5-FU was combined with a nanoscale ultrasound contrast agent using an avidin–biotin interaction. The average particle size, Zeta potential, and concentration of the 5-FU-loaded nanobubbles were measured and analyzed with a Zeta potential instrument (Zetasizer 3000HS; USA). DPPC, DSPE, DPPA, glycerol, and PBS were mixed in appropriate proportions and placed into a 2.0-ml sterile vial. The vial was maintained at 37 °C for 35 min, and a dental amalgamator then was used to shake the vial for 30 s. The mixed materials were incubated under static conditions for 5 min and then rinsed 2 times with PBS, followed by Co^60^ sterilization and storage at 4 °C, resulting in the formation of lipid nanobubbles. Then, the 5-FU was added to these lipid nanobubbles that were manufactured in-house. The average concentration of the 5-FU-loaded nanobubbles was 1.3 × 10^10^/ml; the average diameter was 523.4 ± 104.5 nm, and the average potential of the nanobubbles was −23.2 ± 0.46 mV (*n* = 10). The 5-FU and lipid nanobubbles were effectively combined via the avidin–biotin system, and the combined product is referred to herein as 5-FU-loaded nanobubbles (Fig. 3).

### Treatments

Starting on the seventh day, the 5-FU-loaded nanobubbles were injected into the nude mice via the tail vein once every 2 days for a total of 8 days. Each group received a different treatment as follows: group A, saline (200 µl); group B, 5-FU-loaded nanobubbles (0.1 μg/μl, 200 µl); group C, 5-FU-loaded nanobubbles (0.1 µg/µl, 200 µl) and non-low-frequency ultrasound (diagnostic ultrasound, 2.5 MHz, 2 W/cm^2^) for 5 min; group D, 5-FU-loaded nanobubbles (0.1 μg/μl, 200 µl) and pulse irradiation with low-frequency, focused ultrasound (Ultrasound Research Institute of Chongqing Medical University, 1 MHz, 1.2 W/cm^2^) for 5 min.

### Assessment of therapeutic effect

#### Tumor growth assessment

(A) The status of the hair, nutrition, and tumor metastases of the tumor-bearing nude mice was recorded every 2 days. (B) The inhibition rates were calculated by regularly measuring the tumor size as follows: the maximum short diameter, *a* (cm), and the long diameter, *b* (cm), of each tumor were examined weekly by two-dimensional ultrasound for a total of 4 weeks, and then the tumor volumes in the nude mice were calculated according to the formula *V* = *a* ^2^ *b*/2. Tumor inhibition rate was defined as follows: Tumor inhibition rate = (the average tumor volume in the control group-the average tumor volume in the treatment group)/the average tumor volume in the control group × 100%. Growth curves were generated based on the measured tumor volumes. Additionally, the survival time of each mouse was recorded, and the survival curves were analyzed with the SPSS software.

#### TUNEL staining for the detection of apoptosis in tumor cells

After our treatments, the tumor tissues from some of the nude mice were obtained and sliced into 5 μm sections, deparaffinized with xylene, and then hydrated with an alcohol gradient. The samples were incubated with protease K, 20 μg/ml, for 15 min at 37 °C and then rinsed three times for 5 min each with PBS. The samples were treated with 3% hydrogen peroxide (H_2_O_2_) in methanol for 10 min at room temperature. After washing three times with PBS, 10 μl of TDT and 10 μl of DUTP were added to 1 ml of TUNEL buffer at room temperature for 15 min, and the sections were treated with the TUNEL buffer for 1 h at 37 °C in the dark. The color was developed by exposure to 0.04% DAB and 0.03% H_2_O_2_ for 10 min. Next, the sections were counterstained with hematoxylin contrast dye for 1 min, dehydrated in an increasing gradient of ethanol, washed, and then sealed with conventional resin. Apoptotic cells were counted by two independent investigators using a light microscope with 400 times magnification. Finally, the apoptotic index (AI) was calculated by counting the positive cells in 5 randomly selected areas; (AI = the number of positive tumor cells/the total number of tumor cells × 100%). The criteria for positive cells were that the positive cells were brown and that the staining was located in the cytoplasm of the tumor cells.

#### Detection of tumor vessels and neovascularization with CD34 immunohistochemistry and determination of microvascular density

After treatment, the tumor tissues of some of the nude mice were sliced into 4 μm sections, followed by dewaxing and gradient ethanol hydration. Then, the sections were pretreated with citrate buffer (pH 6.0) using an autoclave for 18 min at 121 °C, after which the sections were cooled naturally and then rinsed three times with PBS for 5 min each. The slices were then treated with 3% H_2_O_2_ for 5 min and rinsed three times with PBS for 5 min each. Next, the sections were immunostained with anti-CD34 antibody (diluted 1:50, Zhongshan Biological Technology, Beijing, China) and were treated with DAB for 15 min in the dark at room temperature. Finally, the sections were washed, counterstained, dehydrated, and mounted. After immunostaining, the CD34-positive microvessels were observed according to the Elivison two-step method. Firstly, the regions of strongest staining (hot spots) were noted using a low-power objective (40–100 times magnification). Secondly, the number of stained vessels was counted using a high-power objective (200–400 times magnification). Average microvessel density (MVD) (aMVD) was calculated by counting the number of CD34-positive microvessels in multiple selected areas (18 areas), which represented the tumor microvascular density.

### Statistical analysis

All the data were analyzed with the SPSS software (Version 21.0, SPSS Ltd., Chicago, Illinois, USA), and the data were expressed as the mean ± standard deviation (SD). The inhibition rate was assessed by an analysis of variance, and the LSD *t* test was applied for pairwise comparisons. The Kaplan–Meier method was used for the analysis of survival. *P* values < 0.05 were considered to be statistically significant.

## Results

### Treatment effects

**Fig. 4:**
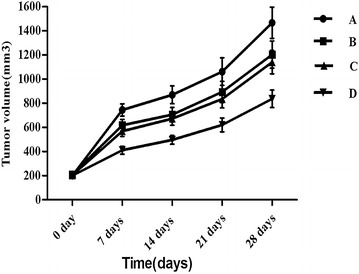
It can be seen from the tumor growth curve that the tumor growth of group D was significantly (*P* < 0.05) slower than that of the other groups

After approximately 2 weeks of treatment, the nude mice in the different groups exhibited a reduction in activity, appetite loss, weight loss, and other symptoms to varying degrees. However, these symptoms were all less in group D (5-FU-loaded nanobubbles with low-frequency ultrasound) than in the other groups. From the tumor inhibition curves (Table 1; Fig. 4), we observed that the tumor volumes in the nude mice in each group gradually increased. However, the tumor growth rate was significantly lower in group D than in the other groups (*P* < 0.05) (Fig. 3; Table 1). The tumor inhibition rates in each group were as follows: A (0%), B (17.38 ± 2.74%), C (22.81 ± 2.67%), and D (43.02 ± 2.54%). Compared with group A, there were statistically significant differences in the other groups; furthermore, there were statistically significant differences between group D and the other groups.

**Table 1:** Comparison of tumor volume after treatment among four groups (*n* = 10, $\overline{x}$  ± s, mm^3^)

Group	After treatment 7 days	After treatment 14 days	After treatment 21 days	After treatment 28 days	Rate of inhibition (%)
A	744.9 ± 51.0	870.5 ± 73.5	1062.3 ± 114.0	1467.2 ± 129.0	0
B	618.6 ± 48.0^△^	706.1 ± 60.4^△^	894.1 ± 88.2^△^	1203.9 ± 112.5^△^	17.38 ± 2.74^△^
C	568.2 ± 42.5^△^	672.4 ± 53.9^△^	835.6 ± 74.1^△^	1141.3 ± 97.1^△^	22.81 ± 2.67^△^
D	411.1 ± 32.6^△^*	496.5 ± 36.1^△^*	620.3 ± 57.3^△^*	836.4 ± 72.0^△^*	43.02 ± 2.54^△^*

Compared with A group, ^△^ *P* < 0.05; compared with C group, * *P* < 0.05

**Fig. 5:**
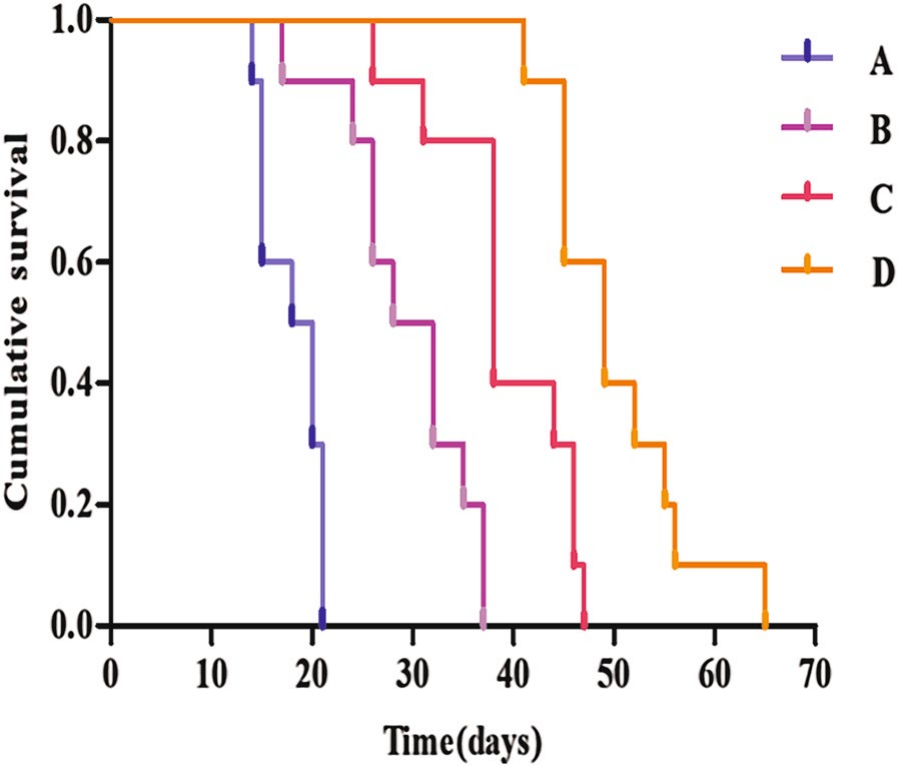
The survival time of tumor-bearing nude mice. From the survival curves we could observe that the survival time of group D was significantly longer than the other groups (*P* < 0.05)

In this study, the survival times of the tumor-bearing nude mice were recorded, and the survival curves were analyzed. Based on the survival times (Fig. 5), we could determine that the survival times of the tumor-bearing nude mice in group D were significantly higher than those in the other groups (*P* < 0.05).

### Apoptosis

**Fig. 6:**
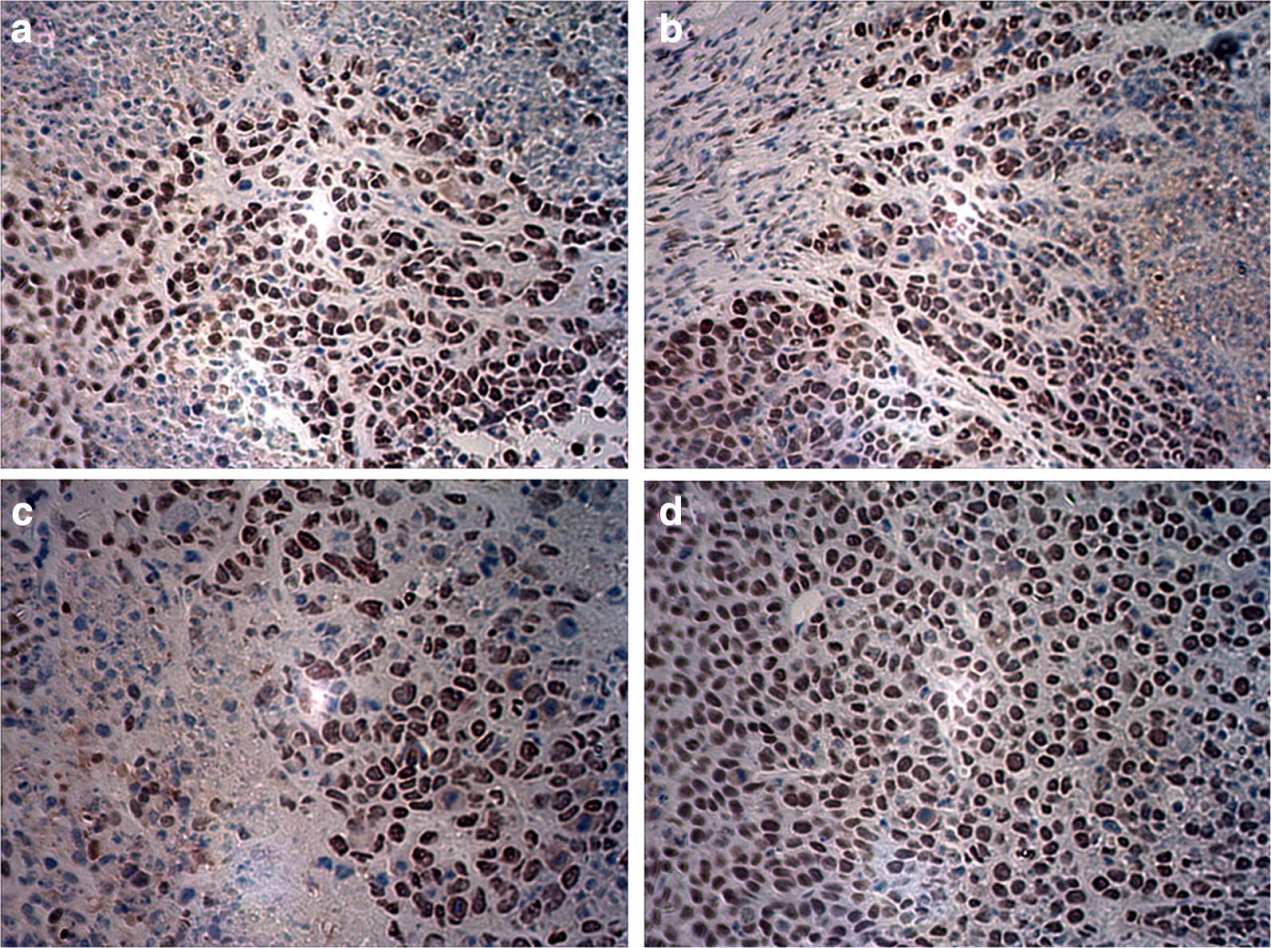
Detection of tumor cell apoptosis with TUNEL staining (original magnification ×400). The number of apoptotic tumor cells in each of the four groups varied (**a** saline; **b** 5-FU-loaded nanobubbles; **c** 5-FU-loaded nanobubbles with non-low-frequency ultrasound; **d** 5-FU-loaded nanobubbles with low-frequency ultrasound)

**Fig. 7:**
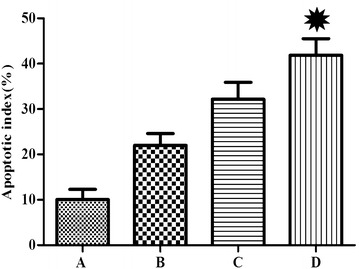
The AI of residual cancer cells in nude mice. The index in group D was significantly higher than that in the other groups (*P < 0.05)

Tumor cell apoptosis was detected by TUNEL staining in each group to confirm that the 5-FU-loaded nanobubbles with low-frequency ultrasound could induce the apoptosis of tumor cells. It was observed that the apoptosis of tumor cells in each group was present to varying degrees, and the AI of group D was higher than in the other groups (*P* < 0.05) (Figs. 6, 7; Table 2).

**Table 2:** The apoptotic index (AI) of residual cancer cells in nude mice ($\overline{x}$  ± s)

Group	A	B	C	D
AI (%)	10 ± 2.2	21 ± 2.6	32 ± 3.7	42 ± 3.7*

D group compared with other groups, * *P* < 0.05

### Assessment of tumor vessels and neovascularization with CD34 immunohistochemistry and microvascular density

**Fig. 8:**
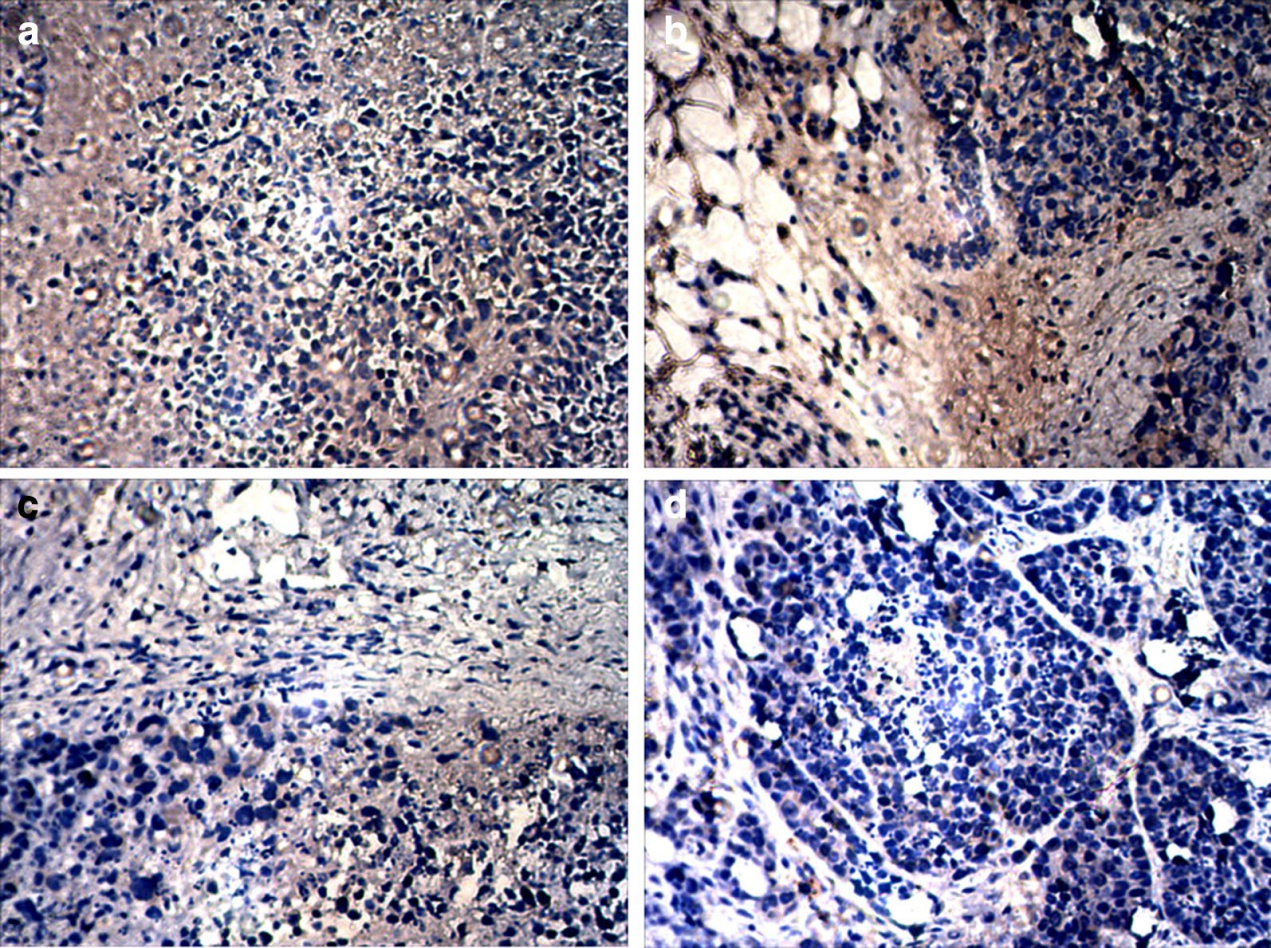
Immunohistochemical staining of CD34 in residual tumor tissues (×200). The number of positively stained areas of tumor tissues in each group varied. (**a** saline; **b** 5-FU-loaded nanobubbles; **c** 5-FU-loaded nanobubbles with non-low-frequency ultrasound; **d** 5-FU-loaded nanobubbles with low-frequency ultrasound)

As depicted in Fig. 8, there were multiple areas with positive staining in the tumor tissues from group A (saline), group B (5-FU-loaded nanobubbles), and group C (5-FU-loaded nanobubbles with non-low-frequency ultrasound). However, there were no areas of obvious positive staining in the tumor tissues of group D. The MVD values of each group were determined according to the CD34-MVD method and were 29.6 ± 4.2, 22.2 ± 3.2, 17.8 ± 3.0, and 7.2 ± 1.5 for groups A, B, C, and D, respectively. The results indicated that the number of tumor vessels and tumor angiogenesis in group D were significantly less than in the other groups (*P* < 0.05).

## Discussion

HCC is a global public health problem, with an incidence 2 to 4 times higher in males than in the females [1, 22]. Additionally, people 35–65 years of age have a high incidence rate [23, 24]. There is also a high prevalence of HCC in Asia and sub-Saharan Africa [25]. In China, HCC is the third most common malignant tumor, second only to gastric and esophageal cancers [2]. Due to the insidious onset of HCC, the early diagnosis of HCC is relatively difficult [4]. As a result, most cases of HCC are already at a moderate or late stage before patients seek medical care. Consequently, those patients can receive only non-operative treatment because they have already progressed past the stage where surgical treatment is an option.

5-FU is a cytotoxic anti-tumor drug with the widest clinical application in the treatment of cancer. 5-FU has a good therapeutic effect in colon, breast, and gastrointestinal cancers, hepatocellular carcinoma, and other cancers [16–18, 26]. A large number of studies have reported that a targeted drug delivery system that uses 5-FU-loaded nanobubbles can selectively transport 5-FU to the site of liver tumors [27, 28], thereby increasing the drug concentration at the tumor site. Additionally, this drug delivery system can slowly release drugs and thus maintain drug concentrations at an effective level for a long period of time [29]. Furthermore, the technology can reduce drug cytotoxicity to normal liver tissues and other organs [30], allowing for lasting and effective anti-tumor effects with a reduction in drug toxicity.

With the rapid development of ultrasound molecular imaging, the UTMD technology is expected to become a new targeted drug delivery technology [5–8, 31]. This technique involves creating lipid microbubbles as drug carriers, which then reach the target tissues via the blood stream after peripheral intravenous injection. The drugs are locally released from the lipid microbubbles after being disrupted by ultrasonic irradiation with extensive energy at the site of accumulation in the target area, which is detected by ultrasonic imaging. Additionally, the “sound hole effect” produced by the ultrasonic irradiation can enhance local tissue microvascular and cell membrane permeabilities, promoting the penetration of drugs and allowing for the targeted release of drugs in vivo to improve the treatment effects [32].

Currently, applications of the UTMD technique primarily rely on ordinary ultrasound (diagnostic ultrasound). This ultrasound technology produces a continuous wave that reduces the perfusion of the microbubbles into the targeted tissue [33]. At the same time, the ultrasound energy can produce a number of biological effects in normal tissues [12–14, 34]. Because the continuous wave cannot be precisely positioned and results in reduced perfusion into the target tissues and organs, the amount of drug that reaches the target tissue is reduced, inhibiting the treatment effects. Before the clinical applications of the drug-loaded microbubbles can be fully realized, technological advances must be made to maximize the local release of the drug while minimizing the damage to normal tissues.

Low-intensity focused ultrasound is based on the same principle of focused ultrasound as that in high-intensity focused ultrasound (HIFU) technology but with much lower energy levels than are used in HIFU [35, 36]. In this study, low-intensity focused ultrasound developed by the Institute of Ultrasound Imaging, Medical University of Chongqing was used as an ultrasound triggering device to disrupt the drug-loaded microbubbles, thereby improving the drug targeting, lowering the toxicity and side effects to normal tissues, and improving the therapeutic effects.

In this study, the growth rate of the tumors in group D (5-FU-loaded nanobubbles with low-frequency ultrasound) was significantly slower than that of the other groups (*P* < 0.05), while the inhibition rate and AI of group D were significantly higher than those of the other groups (*P* < 0.05). The number of microvessels with CD34-positive staining was decreased in group D, and the tumor MVD was significantly lower than that of the other groups (*P* < 0.05). Therefore, group D presented the most significant tumor inhibitory effects.

A possible explanatory mechanism is that the nanoscale microbubbles were disrupted by the “cavitation effect” produced by the low-frequency ultrasound. After the ultrasound exposure, the endothelial cell membranes were damaged and micro-thromboses were formed. Additionally, the tumor-supplying vessels were embolized, and the tumor cells developed local necrosis with the decrease in microvascular density. Additionally, due to the “cavitation effect” generated by the low-frequency ultrasound used to irradiate the nanobubbles, the vascular permeability was increased, promoting 5-FU absorption and providing the 5-FU with easier access to induce the intracellular killing of the HCC cells. This mechanism would explain the increased therapeutic effects we observed, with more apoptosis in the tumor cells, followed by reduced tumor volumes and the control of tumor growth.

In conclusion, our results showed that 5-FU-loaded nanobubbles irradiated with low-frequency ultrasound can significantly reduce the level of CD34 expression in transplanted HCC tumor tissues. This therapeutic technique also reduced the number of microvessels and inhibited the growth of the tumor. Further research of drug-loaded nanobubbles combined with UTMD can thus be expected to deliver a safer, more highly targeted, and more efficient and convenient technology for the localized and controlled-release therapy of HCC.

## Conclusions

In this study, 5-FU-loaded nanobubbles combined with low-frequency ultrasound can further improve targeted drug delivery and effectively inhibit the growth of transplanted tumors, which makes this approach a potentially ideal drug carrier and targeted delivery system. Therefore, this study may lay a basic research foundation for the future treatment of HCC when this technology is combined with other physical methods.

## Abbreviations

DAB: diaminobenzidine
DPPA: diphenylphosphoryl azide
DPPC: dipalmitoylphosphatidylcholine
DSPE: distearoylphosphatidylethanolamine
DUTP: deoxyuridine triphosphate
HIFU: high-intensity focused ultrasound
PBS: phosphate buffer saline
RPMI-1640: Roswell Park Memorial-1640
TDT: terminal deoxynucleotidyl transferase
TUNEL: terminal deoxynucleotidyl transferase dUTP nick end labeling
UTMD: ultrasound-targeted microbubble destruction
